# Comparing image quality of coronary CT angiography with and without ECG-gating in wide-detector CT

**DOI:** 10.3389/fcvm.2025.1570743

**Published:** 2025-04-11

**Authors:** Kun Wang, Yueqiao Zhang, Bin Chen, Hong Ren

**Affiliations:** Department of Radiology, Sir Run Run Shaw Hospital, Zhejiang University School of Medicine, Hangzhou, China

**Keywords:** coronary computed tomography angiography, electrocardiogram, wide-detector CT, image quality, radiation dose

## Abstract

**Objective:**

To compare the image quality, radiation dose, and examination time between non-electrocardiogram (ECG)-gated coronary CT angiography (ECG-less CCTA) and conventional ECG-gated CCTA using wide-detector CT, and validate its clinical applicability.

**Methods:**

In this prospective study, 109 patients with suspected coronary artery disease were divided into ECG-less (Group A, *n* = 59) and ECG-gated (Group B, *n* = 50) groups. Objective metrics (CT attenuation, noise, SNR, CNR), subjective image quality (4-point scale), and examination time were analyzed. Diagnostic performance (sensitivity, specificity) was evaluated against invasive coronary angiography (ICA). A modified ECG-less protocol (Group A2, *n* = 30) was implemented to optimize radiation dose. Plaque characterization agreement was assessed using Cohen’s *κ*.

**Results:**

The ECG-less group demonstrated higher radiation dose (2.83 ± 0.93 vs. 1.90 ± 1.41 mSv, *p* < 0.001) but significantly shorter examination time (225.03 ± 33.37 vs. 330.06 ± 56.35 s, *p* < 0.001). The modified ECG-less protocol reduced the effective dose by 28% (2.03 ± 0.75 mSv, *p* < 0.001 vs. Group A), achieving statistical comparability to the conventional group (*p* = 0.62). Subjective image scores (4-point scale) and SNR/CNR showed no significant differences between groups (*p* > 0.05). ECG-less CCTA achieved per-segment sensitivity/specificity of 93.3%/97.5% and per-patient 94.4%/50% for detecting ≥50% stenosis. Plaque characterization exhibited high agreement (calcified: *κ* = 0.82; non-calcified: *κ* = 0.78; mixed: *κ* = 0.75).

**Conclusion:**

ECG-less CCTA provides comparable image quality and diagnostic accuracy to conventional ECG-gated CCTA while significantly reducing examination time. This technique is applicable in emergency scenarios where ECG lead placement is unfeasible (e.g., severe trauma, unreliable ECG signals).

## 1 Introduction

Coronary Artery Disease (CAD) remains a leading cause of morbidity and mortality worldwide (1, 2). Coronary Computed Tomography Angiography (CCTA), recognized for its non-invasive nature and high negative predictive value, has emerged as an essential tool for quantifying coronary artery stenosis and characterizing atherosclerotic plaques (3–5). Advances in technology have steadily enhanced the success rate and accuracy of CCTA (6). Moreover, there is an increasing demand to broaden its applicability to a wider patient population, address more complex scenarios, boost physicians’ confidence in cardiac diagnostics, and enhance the overall patient experience during examinations.

Nowadays, CCTA is no longer restricted by factors such as patient age, heart rate, and even breath-holding requirements. However, it still relies on electrocardiogram-gated (ECG) scanning (7, 8), which presents certain challenges. While ECG-gated CCTA remains the standard for most patients, ECG-less protocols may fill critical gaps in niche emergency scenarios where ECG lead placement is physically impossible (e.g., severe burns, trauma) or diagnostically unreliable (e.g., cardiac tamponade with attenuated QRS voltage). Additionally, certain patients may be unable to connect to the ECG due to the necessity of cardiac monitoring and various other life-saving measures during the scanning procedure. Furthermore, the need to apply ECG leads can result in patients exposing their chest skin, which negatively impacts the overall patient experience.

With the development of advanced technologies, the latest CCTA scans are equipped with wide detectors (16 cm), high rotation speeds (0.23 s/r), and effective coronary motion correction algorithms. Wide detectors enable single axial scans for coronary imaging, while high rotation speeds achieve superior temporal resolution. Additionally, coronary motion correction algorithms allow software-based compensation for coronary motion. These advancements have paved the way for ECG-less CCTA scanning. Consequently, this study aims to evaluate the differences in imaging performance between ECG-less CCTA and conventional ECG-gated CCTA.

## 2 Materials and methods

### 2.1 Patients

**Figure 1:**
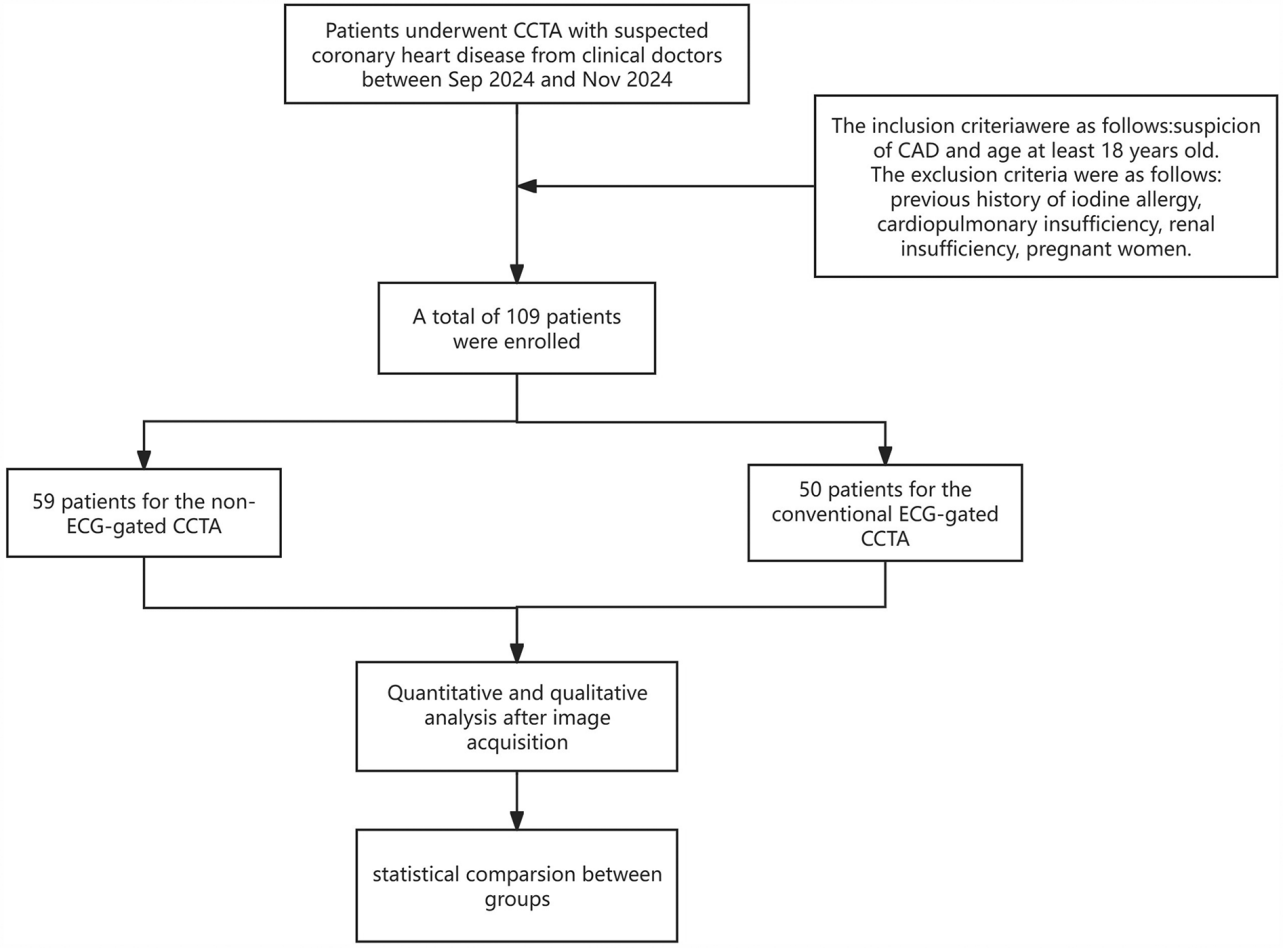
Flow chart of patient enrolment.

This prospective cohort trial was approved by our Institutional Review Board (K2024473) and was conducted following the Declaration of Helsinki (1964). Informed consent was obtained from all patients. We enrolled 109 consecutive patients between July 2024 and November 2024 with suspected CAD undergoing CCTA examination. The first 59 consecutive patients (group A, *n* = 59) were scanned without ECG gating, whereas the remaining consecutive patients (60–109; group B, *n* = 50) were scanned with ECG gating. The inclusion criteria were suspected CAD and age ≥ 18 years. Exclusion criteria were as follows: a history of iodine allergy, a coronary artery bypass graft, cardiopulmonary insufficiency, renal insufficiency and pregnancy. Details of the patient selection process are provided in Figure 1.

Following initial findings, a modified ECG-less protocol was tested in an additional cohort (Group A2, *n* = 30) to explore the feasibility of reducing radiation dose with ECG-less technology. Ethical approval and informed consent were obtained.

### 2.2 Image acquisition and reconstruction

All patients underwent CCTA using a 256-row CT scanner (Revolution Apex Expert, GE Healthcare, WI, USA). For both groups, tube voltage (80, 100, or 120 kV) was automatically selected according to the scout of patients, with automatic tube current modulation and a preset noise index of 20 HU. In group A, ECG-less CCTA was performed, and the data acquisition window was set to 200–750 ms. And in group A2, a narrower time window (300–600 ms) was applied to target diastolic phases (other parameters were kept consistent with group A). A simulated ECG signal was generated based on the estimated heart rate (HR) collected via pulse measurement prior to CCTA, without ECG leads placed on the patient. This simulated ECG signal was used to virtually gate the CT data acquired during a complete heart cycle (9). In group B, a conventional ECG-gated scanning mode was used. The data acquisition window was automatically gated based on the patient’s R-R interval, which was determined by their HR. Threshold monitoring was set at the aortic root, with an enhancement threshold of 180 HU and a delay time of 4 s.

Bolus tracking technique was employed for contrast injection, using Iopromide (370 mgI/100 ml, Bayer, Germany). The contrast volume was calculated as 0.6 ml/kg × body weight, with an injection time of 10 s. This was followed by a saline flush, injected at the same flow rate as the iodine contrast agent for 10 s. Beta-blockers and nitroglycerin were not administered to the patient before the CCTA examination.

Optimal reconstruction phases were automatically selected by the scanning system (Smart Phase technique, GE Healthcare), with a reconstruction slice thickness and interval of 0.625 mm. A coronary motion correction algorithm (SnapShot Freeze 2, SSF2, GE Healthcare) combined adjacent cardiac phases data within a single cardiac cycle to account for and compensate for coronary motion, improving image quality. This technology was specifically applied to vessel segments with significant motion artefacts. All images were reconstructed using deep learning image reconstruction algorithm (TrueFidelity™, GE Healthcare) combined with SSF2, ensuring minimal motion artifacts in the selected phases (10).

### 2.3 Image quality

#### 2.3.1 Objective evaluation

CT attenuations and standard deviations (SD) at the aortic root (AO), proximal left main coronary (LMCA-P), middle left anterior descending artery (LAD-M), distal left anterior descending artery (LAD-D), middle left circumflex branch (LCX-M), distal left circumflex branch (LCX-D), proximal right coronary artery (RCA-P), middle right coronary artery (RCA-M), distal right coronary artery (RCA-D), and perivascular adipose tissue (PVAT) were measured by two cardiac radiologists on axial images on a post-processing workstation (AW4.7, GE Healthcare). The average value obtained from both radiologists was used for further analysis. The SD of the AO was considered image background noise (11). Regions of interest (ROI) were defined within a range of 20–400 mm^2^, carefully avoiding vascular walls and calcified plaque regions to ensure accurate measurements. The signal-to-noise ratio (SNR) and contrast-to-noise ratio (CNR) were calculated using the following equations (12):

(1) SNR = CT attenuation of the ROI/image background noise.
(2) CNR = (CT attenuation of the ROI—CT attenuation of the PVAT)/image background noise.

A coronary artery enhancement threshold of 300 HU was considered satisfactory and sufficiently diagnostic (13).

#### 2.3.2 Subjective evaluation

Image quality was independently evaluated by two radiologists, blinded to the imaging protocol, with 8 years and over 15 years of experience in cardiovascular imaging. The evaluation was based on the 18-segment classification system of the American Heart Association (14). A 4-point Likert scale was used to grade image quality as follows (15): 4 points, all major coronary segments are clearly visualized with adequate luminal enhancement, excellent contrast against surrounding tissues, and no artifacts; 3 points, all major coronary segments are well visualized, though with mild artifacts in the vessel wall or slightly blurred margins, and exhibit fair contrast with surrounding tissues; 2 points, all major coronary segments display moderate or stepped artifacts in the vessel wall and poor contrast but remain diagnostic; and 1 points, all major coronary segments exhibit severe motion artifacts, appear misaligned and discontinuous, and are non-diagnostic.

In addition to stenosis grading, atherosclerotic plaque characterization was performed in a subset of 40 patients (20 from each group) randomly selected from the cohort. Plaques were classified into three categories based on the SCCT guidelines: (1) calcified plaques, ≥130 HU density with no adjacent non-calcified components; (2) non-calcified plaques, <130 HU density without calcification; (3) mixed plaques, Containing both calcified (>130 HU) and non-calcified (<130 HU) components within the same lesion. Two blinded radiologists independently classified plaques on both ECG-less and ECG-gated CCTA images. Inter-observer agreement was calculated using Cohen’s kappa (*κ*) statistics.

### 2.4 ECG-less stenosis diagnosis performance

For the ECG-less group, diagnostic performance was evaluated using invasive coronary angiography (ICA) as the reference standard. Patients who underwent ICA were identified from the ECG-less group through an electronic medical records system. Two experienced observers, with 10 and 15 years of expertise in catheter angiography, independently assessed the degree of vascular stenosis based on ICA images. These observers were blinded to the CCTA results, and any disagreements were resolved through discussion. Similarly, two radiologists with 8 years and over 15 years of experience in cardiovascular imaging independently evaluated the vascular stenosis using CCTA images, with discrepancies also resolved through consensus discussion.

The evaluation of stenosis using ICA and CCTA was conducted at three levels: per-patient level, per-vessel level (four main coronary arteries), and per-segment level (18 segments). We defined significant coronary artery disease as luminal narrowing of ≥50% (16). Segments with a diameter of less than 1.5 mm were excluded from the analysis.

### 2.5 Radiation dose and examination time

The Dose Length Product (DLP) and volume CT dose index (CTDI_vol_) of CCTA imaging for each patient were recorded, and the effective radiation dose (ED) was calculated according to the formula ED = κ* DLP, where *κ* = 0.014 mSv/(mGy × cm) (17). The examination time from lying on the examination bed until the examination was completed was recorded. All examinations were performed by a single technician.

### 2.6 Statistical analysis

The Kolmogorov–Smirnov test was used to evaluate normally distributed data. Normally distributed continuous data are expressed as the mean ± SD, whereas non-normally distributed continuous data are expressed as the median with interquartile range. The count data are expressed as absolute values and corresponding frequencies/percentages. Statistical analysis between two independent samples was performed with the independent sample *t*-test or Mann–Whitney *U*-test. The chi-square test was used for statistical analysis of count data. The consistency of the subjective scores of the two physicians was tested using the kappa value, where 0.21–0.40 indicates poor consistency, 0.41–0.60 indicates moderate consistency, and 0.61–0.80 indicates good consistency. A probability (*p*) value of <0.05 was considered statistically significant. The diagnostic accuracy, sensitivity, specificity, positive predictive value (PPV), and negative predictive value (NPV) of CCTA to detect > 50% diameter stenosis on ICA were calculated from the chi-squared test of the contingency table on the per-segment, per-vessel, and per-patient level. All statistical analyses were performed using IBM SPSS Statistics for Windows, version 26.0. (IBM Corporation, Armonk, NY, USA).

## 3 Results

### 3.1 Patient characteristics

A total of 109 patients were included in the study, with 59 patients in group A and 50 patients in group B. No statistically significant differences were observed in age, height, weight, body mass index, heart rate, gender, clinical history and image noise between the two groups (all *p* > 0.05, Table 1).

**Table 1:** Patient characteristics and iodine contrast media administration.

Characteristics	Group A	Group B	*p* value
Basic information			
Age (years)	62.7 ± 10.9	63.6 ± 12.7	0.689
Female (%)	31 (52.5%)	21 (42.0%)	0.568
Height (m)	1.62 ± 0.07	1.64 ± 0.08	0.162
Weight (kg)	64.35 ± 12.33	63.79 ± 11.15	0.806
Body mass index (kg/m^2^)	24.39 ± 3.78	23.67 ± 3.09	0.244
Heart rate (bpm)	71.47 ± 10.70	67.38 ± 11.95	0.062
Examination time (s)	225.03 ± 33.37	330.06 ± 56.35	<0.001
Volume of ICM (ml)	39.61 ± 8.13	38.96 ± 6.60	0.651
Injection flow rate (ml/s)	3.94 ± 0.77	3.84 ± 0.61	0.466
CTDI_vol_ (mGy)	15.56 ± 5.16	11.06 ± 7.66	<0.001
DLP (mGy·cm)	201.89 ± 66.65	135.41 ± 100.55	<0.001
Effective dose (mSv)	2.83 ± 0.93	1.90 ± 1.41	<0.001
Clinical history			
Hypertension (%)	25.4	28.0	0.682
Hyperlipidemia (%)	20.3	14.0	0.961
Smoking (%)	23.7	26.0	0.081
Diabetes (%)	8.5	10.0	0.260

In terms of radiation dose, the ED was significantly higher in group A than in group B (2.83 ± 0.93 vs. 1.90 ± 1.41 mSv, *p* < 0.001, Table 1). Additionally, there was a significant difference in the total examination time between group A and group B (225.03 ± 33.37 vs. 330.06 ± 56.35 s, *p* < 0.001, Table 1). The mean examination time in group A was reduced by approximately 32% compared to group B. At the same time, there were no significant differences in the amount and flow rate of iodine contrast agent between the two groups (both *p* > 0.05, Table 1).

### 3.2 Image quality

There was no significant difference in CT attenuation values between the two groups (*p* > 0.05; Table 2), but CT attenuation values for LAD-D and RCA-D were significantly higher in group A than in group B (*p* < 0.05, Table 2). The CT attenuation values of each vascular segment in group A were higher than those in group B (Table 2). In terms of SNR values, there was no significant difference between the two groups (*p* > 0.05, Table 2). There were no significant differences in the CNR values of all vessels between the two groups (*p* > 0.05, Table 2), but CNR for LCX-D were significantly higher in group A than in group B (*p* < 0.05, Table 2). The image quality scores were comparable between groups A and B for all vessels (*p* > 0.05, Table 2), however, the scores of LAD was significantly higher in group A than in B (*p* < 0.05, Table 2). Inter-rater reliability of the qualitative image score was good between the two radiologists (*κ* = 0.86).

**Table 2:** Results of the objective and subjective quality analysis.

Parameter	Group A (*n* = 59)	Group B (*n* = 50)	*P* value
CT values (HU)			
AO	553.23 ± 109.13	537.05 ± 103.22	0.431
LMCA-P	511.27 ± 99.09	493.40 ± 89.44	0.329
LAD-M	444.19 ± 104.32	418.70 ± 98.87	0.196
LAD-D	390.21 ± 83.81	337.12 ± 86.71	0.002
LCX-M	449.41 ± 105.21	431.96 ± 100.82	0.381
LCX-D	386.43 ± 92.15	354.51 ± 107.05	0.097
RCA-P	494.99 ± 93.60	465.67 ± 104.41	0.125
RCA-M	460.30 ± 89.90	438.90 ± 106.66	0.258
RCA-D	475.33 ± 101.45	434.50 ± 106.30	0.043
PVAT	−85.2 ± 12.3	−82.7 ± 11.8	0.215
SNR values			
AO	29.27 ± 5.91	30.75 ± 5.78	0.191
LMCA-P	27.14 ± 5.74	28.38 ± 5.94	0.269
LAD-M	23.68 ± 6.24	24.10 ± 5.95	0.722
LAD-D	20.79 ± 5.31	19.59 ± 6.06	0.275
LCX-M	23.84 ± 5.99	24.73 ± 5.99	0.443
LCX-D	50.56 ± 5.41	20.27 ± 5.94	0.791
RCA-P	26.29 ± 5.58	26.79 ± 6.35	0.662
RCA-M	24.50 ± 5.82	25.28 ± 6.11	0.500
RCA-D	25.34 ± 6.30	25.14 ± 6.73	0.878
CNR values			
AO	34.76 ± 6.66	36.35 ± 6.49	0.211
LMCA-P	32.63 ± 6.54	33.99 ± 6.67	0.286
LAD-M	29.17 ± 6.97	29.70 ± 6.53	0.684
LAD-D	26.28 ± 6.10	19.59 ± 6.06	0.382
LCX-M	29.33 ± 6.67	30.33 ± 6.54	0.443
LCX-D	23.57 ± 6.12	20.27 ± 5.94	0.005
RCA-P	31.78 ± 6.31	32.39 ± 6.99	0.631
RCA-M	29.99 ± 6.43	30.88 ± 6.69	0.481
RCA-D	30.83 ± 6.97	30.75 ± 7.38	0.955
Image noise	19.43 ± 4.65	17.97 ± 4.86	0.113
Qualitative image score (4-point scale)			
LAD	4.0 (4.0, 4.0)	4.0 (4.0, 4.0)	0.033
LCX	4.0 (4.0, 4.0)	4.0 (4.0, 4.0)	0.052
RCA	4.0 (4.0, 4.0)	4.0 (4.0, 4.0)	0.737

AO, aortic root; LMCA-P, proximal left main coronary artery; LAD-M, middle left anterior descending; LAD-D, distal left anterior descending; LCX-M, middle left circumflex; LCX-D, distal left circumflex; RCA-P, proximal right coronary artery; RCA-M, middle coronary right artery; RCA-D, distal coronary right artery; PVAT, perivascular adipose tissue; SNR, sign-to-noise ratio; CNR, contrast-to-noise ratio.

### 3.3 ECG-less stenosis diagnosis performance

In group A, 20 patients underwent ICA examination after CCTA imaging, resulting in the analysis and comparison of 80 coronary arteries and 315 segments. The diagnostic accuracy of ECG-less CCTA for the detection of ≥50% stenosis on per-segment, per-vessel, and per-patient level were 96.5%, 88.8% and 90.0%, respectively (Table 3). The sensitivity of ECG-less CCTA for the detection of ≥50% stenosis on per-segment, per-vessel, and per-patient level were 93.3%, 84.0%, and 94.4%, respectively (Table 3). The weighted kappa value for agreement between two independent readers in CCTA was 0.84 and in ICA was 0.94.

**Table 3:** Diagnostic performance of ECG-less CCTA for detection of ≥50% stenosis.

Evaluation level	Per-segment	Per-vessel	Per-patient
Accuracy, %	96.5 (304/315)	88.8 (71/80)	90.0 (18/20)
Sensitivity, % (95% CI)	93.3 (84.7–97.2)	84.0 (63.1–94.7)	94.4 (70.6–99.7)
Specificity, % (95% CI)	97.5 (94.8–98.9)	90.9 (79.1–96.6)	50 (9.5–90.5)
PPV, % (95% CI)	92.1 (83.2–96.7)	84.2 (63.1–94.7)	94.4 (70.6–99.7)
NPV, % (95% CI)	97.9 (95.0–99.1)	92.6 (81.1–97.6)	50.0 (9.5–90.5)

PPV, positive predictive value; NPV, negative predictive value; CI, confidence interval.

The modified ECG-less protocol (Group A2) achieved a 28% reduction in effective dose (2.03 ± 0.75 mSv vs. 2.83 ± 0.93 mSv in Group A, *p* < 0.001) while maintaining diagnostic accuracy (per-segment sensitivity: 91.2% vs. 93.3%, *p* = 0.45; specificity: 96.8% vs. 97.5%, *p* = 0.68). Radiation dose in Group A2 was statistically comparable to conventional ECG-gated CCTA (1.90 ± 1.41 mSv, *p* = 0.62) (Supplementary Table S1).

In a *post hoc* analysis of 40 patients (20 per group), ECG-less CCTA demonstrated high agreement with ECG-gated CCTA in plaque characterization (calcified: *κ* = 0.82; non-calcified: *κ* = 0.78; mixed: *κ* = 0.75) (Table 4).

**Table 4:** Agreement in plaque characterization between ECG-less and ECG-gated CCTA.

Plaque type	Cohen’s *κ* (95% CI)	Agreement level	Number of plaques (ECG-less/ECG-gated)	*P*-value
Calcified plaques	0.82 (0.75–0.89)	Excellent	58/60	<0.001
Non-calcified plaques	0.78 (0.70–0.86)	Good	34/32	<0.001
Mixed plaques	0.75 (0.67–0.83)	Good	28/30	<0.001

## 4 Discussion

This study demonstrated that the objective image quality and subjective scores of ECG-less CCTA were not inferior to those of conventional ECG-gated CCTA, and ECG-less CCTA reduced examination time by approximately 46.6%. However, the radiation dose of ECG-less CCTA was significantly higher than that of conventional. ECG-less CCTA is both feasible and broadly applicable, as it does not impose strict on heart rate, rhythm or respiratory state. These advantages are attributable to advancements in the hardware and software of modern CT technology. On the hardware side, wide-body CT detectors now provide a maximum coverage of up to 16 cm, enabling single axial scans for coronary artery imaging. Additionally, gantry rotation speeds have been increased to as fast as 0.23 s/r, significantly reducing motion artifacts during scanning. From the software perspective, algorithms specifically designed to correct for coronary motion have been developed. Notably, the SSF2 algorithm, which reconstructs coronary arteries using three-phase images, offers enhanced correction for coronary motion, further improving image quality. Moreover, the Smart Optimal Phase Selection Technology (Smartphase) can quickly and automatically select the phase with the least artefacts among multiple phases of the cardiac coronary arteries to reconstruct the CCTA with the best phase, which is an indispensable and critical key in achieving the success of ECG-less CCTA.

In this study, the CT values of all vascular segments in group A were higher than those in group B, and all exceeded 325 HU, meeting the requirements of coronary diagnosis guidelines (16). There were no significant differences between the two groups regarding SNR and image noise. In terms of CNR, the value for LCX-D in group A was significantly higher than that in group B, while no significant difference was observed in the other vascular segments. Regarding the subjective scores, all three main vessels received 4 points in group A, and the image quality fully met the diagnostic requirements. The diagnostic accuracy was 90.0% at the per-patient level in the 20 patients in the present study, which is consistent with these reports in terms of diagnostic accuracy (18, 19). However, only 20 patients (33.9%) in the ECG-less group underwent ICA, introducing potential verification bias. This reflects real-world clinical practice where ICA is typically reserved for high-risk cases. To address this, in future trial, enrolling only patients who have both CCTA and ICA.

Beyond stenosis grading, our study demonstrates that ECG-less CCTA achieves high accuracy in plaque characterization (*κ* = 0.82 for calcified plaques), comparable to ECG-gated protocols. This capability is critical for risk stratification, as vulnerable non-calcified plaques are associated with higher cardiovascular event rates (3, 5). The slightly lower agreement for mixed plaques (*κ* = 0.75) may reflect challenges in delineating heterogeneous components during rapid coronary motion, which could be mitigated by future motion correction algorithms.

In this study, the average effective radiation dose of ECG-less CCTA was 2.83 ± 0.93 mSv, which increased by about 48% compared with conventional prospectively ECG-gated CCTA. However, compared with other studies, the effective radiation dose of ECG-less CCTA is close to or even lower than that of the third-generation dual-source CT (20). The slightly higher effective radiation dose in this study was mainly due to the widened acquisition time window (200–750 ms). To address this issue, upcoming research could investigate these dose optimization strategies: (1) Dynamic Tube Current Modulation, which entails adjusting the tube current in real-time according to vascular motion patterns during the card.ac cycle, thereby minimizing unnecessary radiation exposure during high-motion phases (21); (2) AI-Driven Reconstruction Algorithms, where deep learning methods such as TrueFidelity™ effectively reduce image noise at lower radiation doses, improving the signal-to-noise ratio (11); (3) Personalized Acquisition Windows, where narrowing the time window to 300–500 ms for patients with stable heart rates helps capture essential diastolic phases while also decreasing radiation dose (7).

In a follow-up study, we found that our modified ECG-less protocol achieved a 28% reduction in effective dose (2.03 mSv) compared to the original protocol, approaching the dose level of conventional ECG-gated CCTA (1.90 mSv, *p* = 0.62, Supplementary Table S1). This was accomplished through a combination of narrowed acquisition windows (300–600 ms).

**Figure 2:**
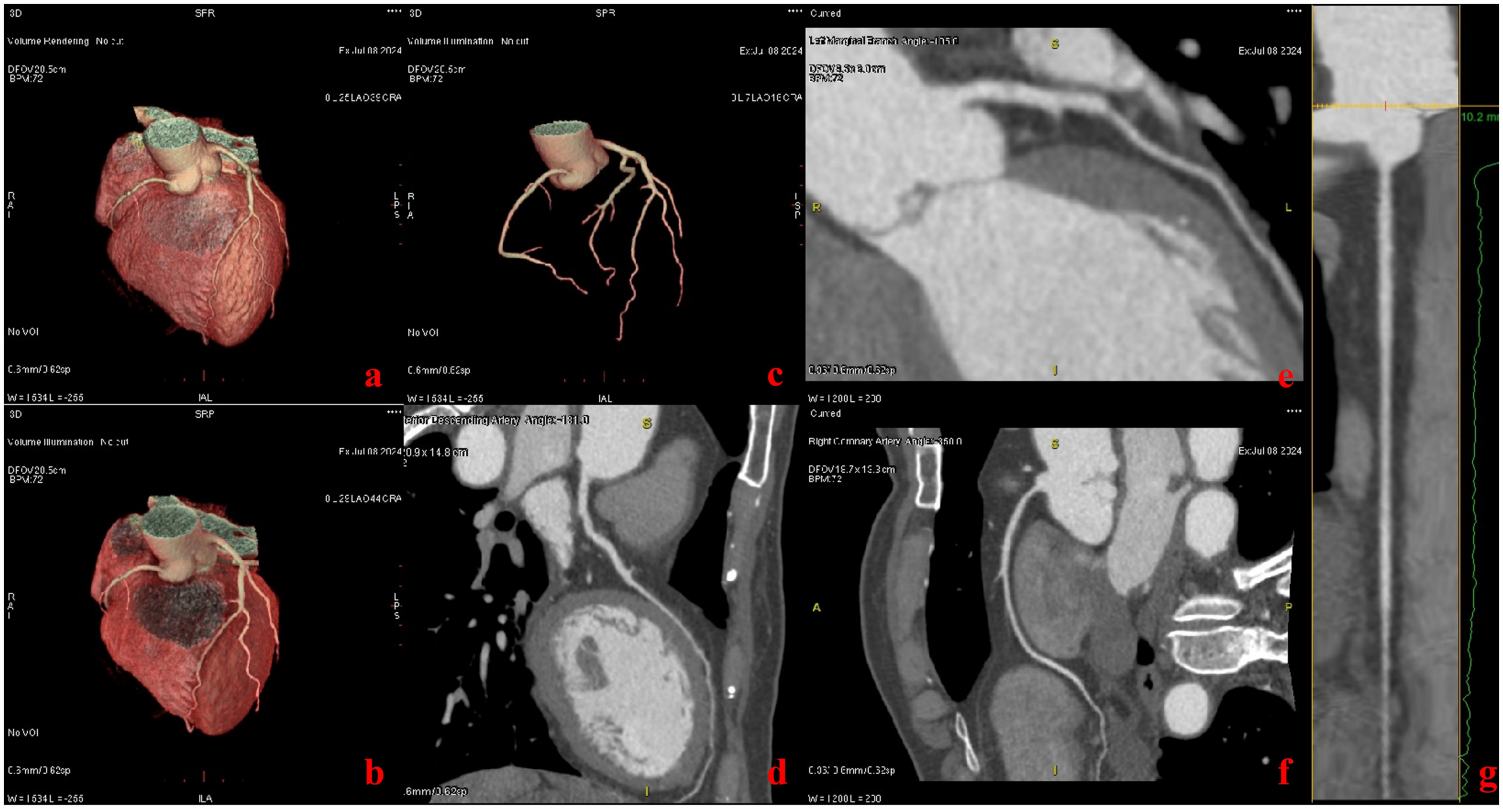
Patient with chief complaint of chest pain (heart rate: 68 bpm). Images were acquired with the ECG-less CCTA. Volume rendering reformat CT images show the heart **(a–c)**. Curved multiplanar reformat CT images show the left anterior descending artery **(d)**, left marginal branch **(e)**, and the right coronary artery **(f, g)**.

Two studies (21, 22) showed that prospectively ECG-triggered CCTA in a single cardiac cycle can achieve high image quality and accuracy with low radiation dose using a wide detector and single-source CT system, but it was limited to patients with HRs <75 beats/min. In this study, we found that ECG-less CCTA can obtain satisfactory image quality between 51 bpm and 112 bpm (Table 1, Figure 2). Therefore, we can assume that ECG-less CCTA can maintain image quality at arbitrary heart rates.

The average examination time for ECG-less CCTA is 225 s (Table 1), allowing for the completion a full CCTA in four minutes while maintaining satisfactory image quality. The mean time reduction of 1.7 min with ECG-less CCTA may appear modest in routine practice. Its value is most evident in scenarios where ECG lead placement is precluded, such as: (1) severe thoracic trauma or burns preventing lead adhesion; (2) cultural/religious objections to chest exposure; (3) Unreliable ECG signals (e.g., cardiac tamponade, hyperkalemia). In these cases, ECG-Less gating eliminates delays associated with lead application or signal optimization.

In this study, we have demonstrated that ECG-less CCTA can reduce examination time and improve the efficiency of CCTA while maintaining image quality. However, this study has the following limitations. First, the relatively small sample size, particularly the subgroup of patients undergoing ICA for validation (*n* = 20 in the ECG-less group), may have introduced statistical instability in specificity calculations. For instance, a single false-positive case disproportionately reduced per-patient specificity (50%), underscoring the need for larger validation cohorts. Second, subgroup analyses based on heart rate, BMI, or coronary calcification burden were not feasible due to imbalanced distributions (e.g., only 5 patients with BMI > 30 kg/m^2^ in the ECG-less group), which limits insights into performance variability across clinically relevant subpopulations. To address these limitations, we will conduct a multicenter trial with protocol- mandated ICA validation for all participants in the future. This initiative will enable robust subgroup stratification and refine the technique’s applicability in complex populations, including those with high calcium burden or arrhythmias. Third, the ECG-less CCTA radiation dose is higher than the conventional ECG-gated CCTA radiation dose because of the wide exposure interval (200–750 ms) we set. Therefore, future research should reduce the exposure time without affecting the conclusions of this study. Fourth, the single-center recruitment and subjective image quality assessments by institutional radiologists may introduce selection and observer bias. To enhance external validity, future studies will involve blinded image evaluations by independent readers from external centers. At last, emergency patients were excluded from this study, limiting direct extrapolation of our findings to time-sensitive scenarios. This design choice was made to ensure protocol standardization, but future trials must validate ECG-less CCTA in hemodynamically unstable cohorts.

## 5 Conclusion

In conclusion, ECG-less CCTA can obtain satisfactory image quality while shortening the examination time and improving the examination efficiency. Furture multicenter, large-scale studies are warranted to further validate its clinical value in complex lesions and acute/critical care scenarios.

## Data Availability

The original contributions presented in the study are included in the article/Supplementary Material, further inquiries can be directed to the corresponding author.
