# Supplementary material for: Comparing image quality of coronary CT angiography with and without ECG-gating in wide-detector CT
Source: Front Cardiovasc Med. 2025 Apr 11;12:1570743. doi: 10.3389/fcvm.2025.1570743 (PMC12021873; doi:10.3389/fcvm.2025.1570743)
Supplement: Supplementary file 1 [file Table1.docx]

|  | ECG-Less Group (Group A) | Modified ECG-less protocol (Group A2) | ECG-gated Group (Group B) | *P*-Value (A vs. B) | *P*-Value (A2 vs. B) |
| --- | --- | --- | --- | --- | --- |
| ED (mSv) | 2.83 ± 0.93 | 2.03 ± 0.75 | 1.90 ± 1.41 | < 0.001 | 0.62 |
| DLP (mGy*cm) | 201.89 ± 66.65 | 145.30 ± 50.20 | 135.41 ± 100.55 | < 0.001 | 0.55 |
| Per-segment sensitivity (%) | \| 93.3 (84.7–97.2) \| \| --- \| | 91.2 (82.5–95.8) | 94.0 (85.1–97.9) | 0.71 | 0.45 |
| Per-segment specificity (%) | 97.5 (94.8–98.9) | 96.8 (93.1–98.5) | 97.8 (95.0–99.0) | 0.82 | 0.68 |
| Per-patient specificity (%) | 50.0 (9.5–90.5) | 55.6 (21.2–86.3) | 52.0 (10.5–89.5) | 0.91 | 0.88 |
| Examination time ( s) | 225.03 ± 33.37 | 220.50 ± 30.15 | 330.06 ± 56.35 | 0.001 | < 0.001 |

Table S1. Comparison of radiation dose and diagnostic performance between the modified ECG-less protocol and the control group.

ED, effective dose; DLP, dose length product.
